# Longitudinal Analysis Is More Powerful than Cross-Sectional Analysis in Detecting Genetic Association with Neuroimaging Phenotypes

**DOI:** 10.1371/journal.pone.0102312

**Published:** 2014-08-06

**Authors:** Zhiyuan Xu, Xiaotong Shen, Wei Pan, for the Alzheimer's Disease Neuroimaging Initiative

**Affiliations:** 1 Division of Biostatistics, School of Public Health, University of Minnesota, Minneapolis, Minnesota, United States of America; 2 School of Statistics, University of Minnesota, Minneapolis, Minnesota, United States of America; The University of Chicago, United States of America

## Abstract

Most existing genome-wide association analyses are cross-sectional, utilizing only phenotypic data at a single time point, e.g. baseline. On the other hand, longitudinal studies, such as Alzheimer's Disease Neuroimaging Initiative (ADNI), collect phenotypic information at multiple time points. In this article, as a case study, we conducted both longitudinal and cross-sectional analyses of the ADNI data with several brain imaging (not clinical diagnosis) phenotypes, demonstrating the power gains of longitudinal analysis over cross-sectional analysis. Specifically, we scanned genome-wide single nucleotide polymorphisms (SNPs) with 56 brain-wide imaging phenotypes processed by FreeSurfer on 638 subjects. At the genome-wide significance level (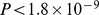) or a less stringent level (e.g. 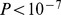), longitudinal analysis of the phenotypic data from the baseline to month 48 identified more SNP-phenotype associations than cross-sectional analysis of only the baseline data. In particular, at the genome-wide significance level, both SNP rs429358 in gene APOE and SNP rs2075650 in gene TOMM40 were confirmed to be associated with various imaging phenotypes in multiple regions of interests (ROIs) by both analyses, though longitudinal analysis detected more regional phenotypes associated with the two SNPs and indicated another significant SNP rs439401 in gene APOE. In light of the power advantage of longitudinal analysis, we advocate its use in current and future longitudinal neuroimaging studies.

## Introduction

**Figure 1:**
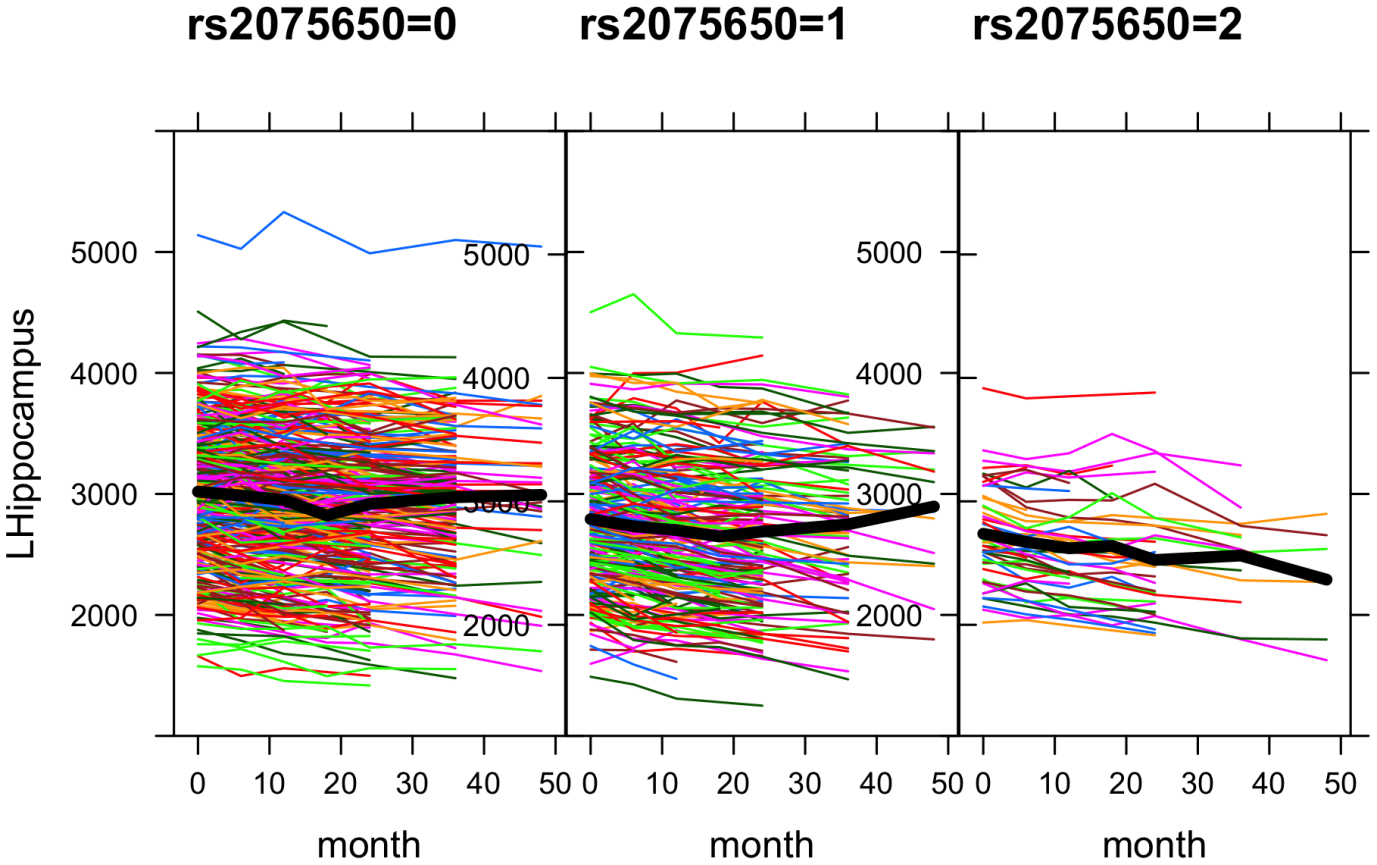
Trajectories of phenotype left hippocampus volume over time (in months) in three allele groups of SNP rs2075650.

There has been increasing interest in genome-wide association studies (GWASs) with neuroimaging phenotypes. Alzheimer's Disease Neuroimaging Initiative (ADNI) provides a rich source of brain imaging, neuropsychological and genetic data, including genome-wide single nucleotide polymorphisms (SNPs) [1], [2]. In ADNI (or more specifically ADNI-1), while the subjects were followed up to 5 years, most of the previous GWAS analyses of brain-wide imaging phenotypes ignored the longitudinal data and mainly focused on only the baseline phenotypes [3]–[9]. In genome-wide association studies longitudinal analysis has been proposed and applied [10]–[14], and in particular its advantage over cross-sectional analysis has been established [15]. Hence, instead of using only the baseline structural MRI scans as phenotypes, we took advantage of the longitudinal imaging phenotypes measured at multiple time points from the baseline to 48 months, demonstrating the application of a linear mixed-effects model and its associated power gains. The advantage of longitudinal analysis is not surprising: assuming no SNP-age interactions, a cross-sectional study based on the baseline can only capture the mean differences of a phenotype across the (genetic) subgroups of subjects; in contrast, a longitudinal study offers the opportunity to estimate not only the mean values of the phenotype at the baseline, but also the rates of the changes of the phenotype in the genotypic groups. For example, as shown in Figure 1, the trajectories of the hippocampal volume appear to decline much faster for the subjects with the homozygotic minor alleles of SNP rs2075650 in gene TOMM40 than those from other two genotype groups. However, we also notice the variations in the rates (i.e. slopes) of the changes across the subjects, which call for a suitable statistical model to account for this source of variations. As to be shown, some alternative but popular and simpler models would fail for the longitudinal data here.

## Materials and Methods

### Data

Data used in the preparation of this article were obtained from the Alzheimer's Disease Neuroimaging Initiative (ADNI) database (adni.loni.usc.edu). The ADNI was launched in 2003 by the National Institute on Aging (NIA), the National Institute of Biomedical Imaging and Bioengineering (NIBIB), the Food and Drug Administration (FDA), private pharmaceutical companies and non-profit organizations, as a $60 million, 5-year public-private partnership. The primary goal of ADNI has been to test whether serial magnetic resonance imaging (MRI), positron emission tomography (PET), other biological markers, and clinical and neuropsychological assessment can be combined to measure the progression of mild cognitive impairment (MCI) and early Alzheimer's dementia (AD). Determination of sensitive and specific markers of very early AD progression is intended to aid researchers and clinicians to develop new treatments and monitor their effectiveness, as well as lessen the time and cost of clinical trials.

The Principal Investigator of this initiative is Michael W. Weiner, MD, VA Medical Center and University of California-San Francisco. ADNI is the result of efforts of many co-investigators from a broad range of academic institutions and private corporations, and subjects have been recruited from over 50 sites across the U.S. and Canada. The initial goal of ADNI was to recruit 800 subjects but ADNI has been followed by ADNI-GO and ADNI-2. To date these three protocols have recruited over 1500 adults, ages 55 to 90, to participate in the research, consisting of cognitively normal older individuals, people with early or late MCI, and people with early AD. The follow up duration of each group is specified in the protocols for ADNI-1, ADNI-2 and ADNI-GO. Subjects originally recruited for ADNI-1 and ADNI-GO had the option to be followed in ADNI-2. For up-to-date information, see www.adni-info.org.

Specifically, we started with the following data on 818 subjects in ADNI-1 [2]: FreeSurfer-processed brain imaging phenotypes, Illumina SNP genotypes and demographic information (including handedness, years of education, gender and age). For data quality control, following [4]. we adopted the following procedure: (1) including only non-Hispanic Caucasians; (2) checking each subject's identity and gender; (3) excluding the subjects with heavy missing values. At the end, we had 638 subjects remaining in the study.

The phenotypic data were processed with the FreeSurfer image analysis suite [16] by UCSF researchers [17]. Briefly, FreeSurfer version 4.3 was applied to T1 weighted structural MRI in the NiFTI format after being pre-processed by the Mayo Clinic [1]. Both longitudinal and cross-sectional registrations were used for the corresponding longitudinal and cross-sectional analyses. For cross-sectional processing, each scan was segmented according to an atlas defined by FreeSurfer, allowing for comparison between groups at a single time point. For longitudinal processing, for each subject with images at more than one time point, a within-subject template based on his/her average image was created using robust inverse consistent registration [18]. Then each subject's template was used to initialize the longitudinal image processing to increase the reliability and statistical power when measuring the brain changes over time [18].

For cross-sectional analysis, we used the phenotypic data only at the baseline, while for longitudinal analysis we used all the data for each subject, up to the measurements at the other five time points (months 6, 12, 24, 36 and 48) beyond the baseline. Since many subjects were not measured at some time points, we used only available data without imputation for either phenotypic or genotypic data.

### Statistical models

For cross-sectional analysis of the baseline data, we use a (standard) linear regression model:
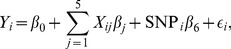
(1)for subject 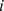, 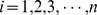. 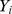 is the (regional imaging) phenotype of subject 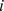 at the baseline; 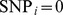, 1 or 2 is the count of the minor allele for the SNP to be tested; 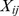 is one of the five covariates: left or right handedness, education in years, age at the baseline, gender and the baseline intracranial volume (ICV); and 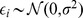 is an independent error term. The goal is to test the null hypothesis 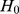: 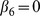 versus 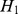: 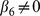. We conduct single SNP-based analysis on each phenotype: each of the SNPs is tested one by one on each regional imaging phenotype sequentially.

For longitudinal analysis, we use a linear mixed-effects model with a random slope and a random intercept [19]:
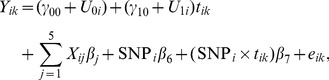
(2)for 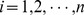 and 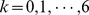, where 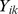 is the (regional imaging) phenotype value of subject 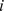 at time point 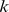, 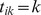 is the time point 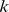, 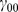 and 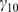 are the fixed intercept and fixed slope for time, and 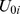 and 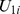 are random intercept and slope respectively:



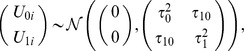
and 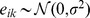 is an independent error term. Other terms are the same as in the standard linear regression model. The two random terms induce some complex and time-varying within-subject correlations and variances: for 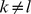, we have



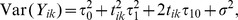


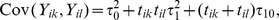
each of which is a function of time unless 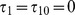.

The goal in the longitudinal analysis is to test for no joint main- and interaction-effects of the SNP with 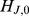: 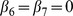 versus 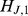: 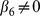 or 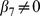. This is the default test to be used in longitudinal analysis. Although the above test is preferred [19], sometimes we would like to see the separate contributions of the main effects of the SNP and the SNP-time interaction, thus we would conduct two separate tests. The first is to test for the zero main effects of the SNP with 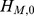: 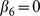 versus 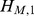: 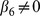, and the second is for the zero SNP-time interaction effects with 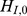: 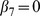 versus 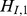: 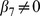.

The purpose of introducing the random intercept and slope parameters is to take account of likely within-subject correlations among the multiple phenotypes for the same subject; in addition, the random slope parameter can account for heterogeneity of the slope parameters among the subjects. Figure 1 shows the trajectories of an imaging phenotype, volume of left hippocampus, for the three groups of the subjects with various genotypes of SNP rs2075650. It is clear that the longitudinal values of the imaging phenotype at the several time points for the same subject are more or less similar to each other as compared to the values from a different subject, suggesting the necessity of using the random subject-specific intercept term 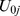; on the other hand, the rates of the change of the phenotype over time for different subjects may be different, implying the use of the random subject-specific slope parameter 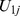. We used the function lme ( ) in R package nlme to fit the linear mixed-effects model.

### Alternative models for longitudinal analysis

A simpler linear mixed model contains only a random intercept term, which is perhaps more commonly used for longitudinal data:
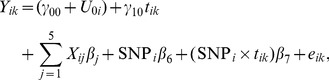
(3)for which the parameters are specified as in model (2). Compared to model (2), the random slope parameter 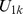 is missing in the new model (3). It is easy to verify that both models share the following mean function of the phenotype (conditional on the covariates):



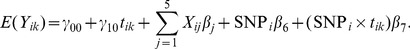
(4)However, their variances are different: instead of having a time-varying within-subject covariance matrix for model (2), we have
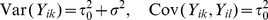
(5)for 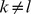 for model (3), suggesting a within-subject compound symmetry (CS) correlation matrix. Obviously model (3) is a special case of model (2).

To compare model (3) against model (2), we can test the null hypothesis 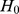: 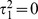 via a likelihood ratio test (LRT). The null distribution of the LRT statistic 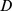 can be approximated by a mixture distribution, 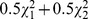 [20].

Alternatively, rather than using a linear mixed model, we can use generalized estimating equations (GEE) to draw inference for longitudinal data [21], [22]. GEE only requires to correctly specify a mean model and use a working within-subject correlation structure. Here we will use the mean model specified in (4), and use a working correlation matrix with a CS structure specified in (5). If a model-based covariance matrix is used, the validity of the GEE results depends on the correct specification of the working correlation structure; if a so-called sandwich or robust covariance matrix is used, then any working correlation can be used. We also note that, in the presence of missing phenotypes, the validity of GEE depends on the assumption of “missing completely at random” (MCAR), a stronger assumption than that of “missing at random” (MAR) required by linear mixed models; this may have implications in real data analysis in the presence of missing phenotypes, which is the case here. We used function geese ( ) from R package geepack to fit a GEE model.

### Simulation set-ups

A longitudinal phenotype 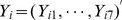 for each subject 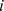 was simulated from the linear mixed-effects model with a random slope and a random intercept as specified in equation (2). Each simulated dataset consisted of 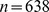 subjects. The phenotypes were generated either under 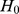 (i.e. 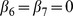) to investigate Type I errors or under 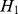 (i.e. 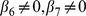) for power analysis; all parameters used were at at the maximum likelihood estimates of model (2) fitted to the original ADNI data. The same set of covariates (i.e. left or right handedness, education in years, age at the baseline, gender and ICV) were used.

To evaluate the robustness of model (2), we also simulated phenotypic data 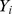 from model (3). The parameters used under 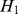 (i.e. 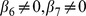) were half of the maximum likelihood estimates in model (3) fitted to the original ADNI data.

Three most significant SNPs in Table 1, rs429358, rs2075650 and rs439401, were chosen to be used in simulations. Under each simulation set-up, 1000 sets of phenotypic data were independently generated and analyzed by five methods: a linear mixed-effects model with both a random slope and a random intercept (LMR-RSI); a linear mixed-effects model with only a random intercept term (LMR-RI); a marginal GEE model with a CS working correlation matrix and with the sandwich covariance estimator (GEE-Robust); a marginal GEE model with a CS working correlation matrix and with the model-based covariance estimator (GEE-Naive); a standard linear model for cross-sectional analysis at the baseline, testing for the main effects of an SNP (Baseline). We then estimated the empirical Type I error rate under 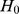 and empirical power under 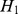 for each method.

**Table 1:** Significant SNPs and each one's associated phenotype numbers at the significance level of 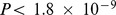.

	Gene		# Phenotypes	
SNP	(Chr)	Position	Longitudinal	Baseline
rs2075650	TOMM40	45,395,619	3	1
	(19)		LHippVol: 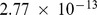	LHippVol: 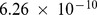
			RCerebCtx: 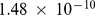	
			LMeanTemp: 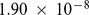	
rs439401	APOE	45,414,451	1	0
	(19)		LMeanLatTemp: 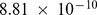	
rs429358	APOE	45,411,941	42	4
	(19)		LHippVol: 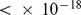	LHippVol: 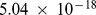
			LEntCtx: 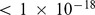	RHippVol: 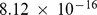
			LAmygVol: 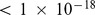	LAmygVol: 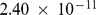
-	-	**total**	46	5

Top 3 SNP-phenotype associations are listed with corresponding P-values.

## Results

### Data summary

After quality control, there were 638 subjects remaining in both cross-sectional and longitudinal data, though there were missing data for some phenotypes and in later follow-ups; see Table 2 for an example. Table 3 summarizes demographic information and intracranial volume (ICV) of the 638 subjects at the baseline. The P-values were calculated based on an F-test for a continuous variable or a Chi-squared test for a categorical variable for its mean or distributional differences among three diagnostic groups at the baseline: healthy normal subjects (HC), subjects with mild cognitive impairment (MCI) and patients with Alzheimer's dementia (AD). The table shows that the distributions of gender and mean years of education were significantly different among the three groups at the significance level 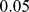.

**Table 2:** The number (percentage) of non-missing observations at each time point in Figure 1.

Month	0	6	12	18	24	36	48
#Obs	635 (99.5%)	616 (96.6%)	574 (90%)	246 (38.6%)	462 (72.4%)	263 (41.2%)	56 (8.8%)

**Table 3:** The baseline characteristics of 638 subjects, including gender, age, years of education, handedness (R/L) and intracranial volume (ICV).

Name	HC	MCI	AD	P-value
number of subjects	182	311	145	-
Gender(M/F)	103/79	204/107	80/65	0.0446
Baseline age	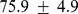	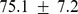	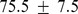	0.4153
Education (years)	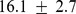	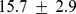	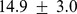	0.0005
Hand(R/L)	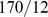	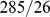	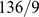	0.6392
ICV	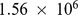	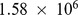	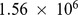	0.1463
	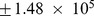	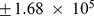	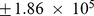	

P-values were calculated to test for differences among the diagnostic groups, HC, MCI and AD.

We conducted cross-sectional and longitudinal analyses of each of about 500,000 SNPs with each of 56 neuroimaging phenotypes in some regions of interest (ROIs) as shown in Table 4 [4].

**Table 4:** 56 cortical thickness and volumetric phenotypes.

Trait Name	Trait Description	Trait Name	Trait Description
AmygVol	Volume of amygdala	MidTemporal	Thickness of middle temporal gyrus
CerebCtx	Volume of cerebral cortex	Parahipp	Thickness of parahippocampal gyrus
CerebWM	Volume of cerebral white matter	PostCing	Thickness of posterior cingulate
HippVol	Volume of hippocampus	Postcentral	Thickness of postcentral gyrus
InfLatVent	Volume of inferior lateral ventricle	Precentral	Thickness of precentral gyrus
LatVent	Volume of lateral ventricle	Precuneus	Thickness of precuneus
EntCtx	Thickness of entorhinal cortex	SupFrontal	Thickness of superior frontal gyrus
Fusiform	Thickness of fusiform gyrus	SupParietal	Thickness of superior parietal gyrus
InfParietal	Thickness of inferior parietal gyrus	SupTemporal	Thickness of superior temporal gyrus
InfTemporal	Thickness of inferior temporal gyrus	Supramarg	Thickness of supramarginal gyrus
MeanCing	Mean thickness of caudal anterior	TemporalPole	Thickness of temporal pole
	cingulate, isthmus cingulate, posterior		
	cingulate, and rostral anterior cingulate		
MeanFront	Mean thickness of caudal midfrontal	MeanTemp	Mean thickness of inferior temporal,
	rostral midfrontal, superior frontal,		middle temporal, superior temporal,
	lateral orbitofrontal, and medial		fusiform, parahippocampal, ling-
	orbitofrontal gyri and frontal pole		ual gyri temporal pole and transverse
			temporal pole
MeanLatTemp	Mean thickness of inferior temporal,	MeanSensMotor	Mean thickness of precentral and
	middle temporal, and superior		postcentral gyri
	temporal gyri		
MeanMedTemp	Mean thickness of fusiform,	MeanPar	Mean thickness of inferior and
	parahippocampal, and lingual gyri,		superior parietal gyri, supramarginal
	temporal pole and transverse		gyrus, and precuneus
	temporal pole		

There are 2 phenotypes for each given phenotype name at the left and right sides of the brain respectively.

Figure 1 shows the longitudinal values of one phenotype, left hippocampus volume, for subjects in each of the three genotype groups for a (significant) SNP. We note the variation of the slope of the subject-specific phenotype trajectory, which calls for the use of a random slope parameter as specified in linear mixed model (2). We also note many missing values at some time points (Table 2); our analyses were all based on observed phenotypic (and genotypic) data without imputation.

### Genome-wide association testing: a summary

Since a total number of 56 phenotypes were to be tested, taking the usual genome-wide significance level for a single phenotype at 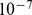 (or 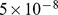), we used the Bonferroni method for multiple testing correction, yielding the significance cut-off at 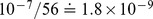 (or 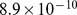).

Under the significance level 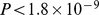, a total number of 46 pairs of significant SNP-phenotype associations were detected in longitudinal analysis, including three SNPs, rs429358, rs2075650 and rs439401 that were associated with 3, 1 and 42 out of the total 56 phenotypes respectively (Table 1). In contrast, only 5 significant SNP-phenotype association pairs were detected in cross-sectional analysis at the baseline, involving only two SNPs, rs2075650 and rs429358, which were associated with only 1 and 4 phenotypes respectively. We reached the same conclusion with a more stringent significance level 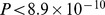, as shown in Table 5.

**Table 5:** Significant SNPs and each one's associated phenotype numbers at the level of 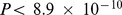.

	Gene		# Phenotypes	
SNP	(Chr)	Position	Longitudinal	Baseline
rs2075650	TOMM40	45,395,619	2	1
	(19)		LHippVol: 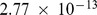	LHippVol: 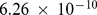
			RCerebCtx: 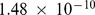	
rs439401	APOE	45,414,451	1	0
	(19)		LMeanLatTemp: 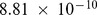	
rs429358	APOE	45,411,941	40	4
	(19)		LHippVol: 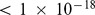	LHippVol: 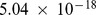
			LEntCtx: 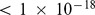	RHippVol: 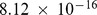
			LAmygVol: 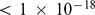	LAmygVol: 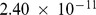
-	-	**total**	43	5

Top 3 SNP-phenotype associations are listed with corresponding P-values.

If a less stringent significance level was employed, an even more substantial difference between the results of longitudinal analysis and that of cross-sectional analysis emerged. As shown in Table 6, at 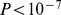, seven SNPs, rs2075650, rs4902433, rs439401, rs11762610, rs1800627, rs11677350 and rs429358, were discovered to be associated with some phenotypes in longitudinal analysis; in contrast, only five SNPs, rs2075650, rs11677350, rs429358, rs2931352 and rs11875359 were detected from cross-sectional analysis. The same conclusion was reached with even a less stringent 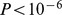 (not shown): one hundred and forty-seven significant SNP-phenotype association pairs were identified from longitudinal analysis, while only 47 pairs were found from cross-sectional analysis.

**Table 6:** Significant SNPs and each one's associated phenotype numbers at the level of 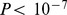.

SNP	L	I	M	B
rs2075650	25	12	3	1
	LHippVol: 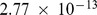			LHippVol: 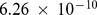
	RCerebCtx: 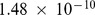			
	LMeanTemp: 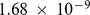			
rs11677350	1	0	1	1
	RCerebWM: 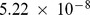			RCerebWM: 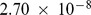
rs4902433	2	0	0	0
	LMeanLatTemp: 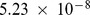			
	LInfTemporal: 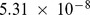			
rs439401	6	10	0	0
	RMeanLatTemp: 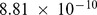			
	RcerebCtx: 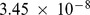			
	RMeanTemp: 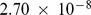			
rs11762610	2	0	0	0
	LFusiform: 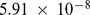			
	LInfTemporal: 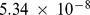			
rs1800627	1	0	0	0
	RAmygVol: 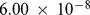			
rs429358	46	40	5	5
	LHippVol: 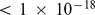			LHippVol: 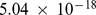
	LEntCtx: 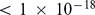			RHippVol: 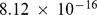
	LAmygVol: 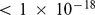			LAmygVol: 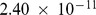
rs2931352	0	0	0	1
				RParahipp: 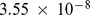
rs11875359	0	0	0	1
				RInfLatVent: 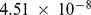
**total**	**83**	**62**	**9**	**9**

In longitudinal analysis, column name “L”, “I” and “M” indicate the number of traits associated with the SNP from the longitudinal joint testing (i.e. with 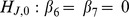), testing for interaction (i.e. 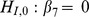) and testing for the main effects (i.e. 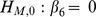); the column named “B” is for cross-sectional analysis of the baseline data. Top 3 SNP-phenotype association are listed with corresponding P-values.

In summary, both longitudinal and cross-sectional analyses indicated that both left and right hippocampus and amygdala were statistically significantly associated with SNP rs429358 in gene APOE at the genome-wide significance level 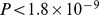. In addition, SNP rs2075650 in gene TOMM40 was also identified to be associated with multiple phenotypes. These results are in agreement with some earlier studies. For example, both TOMM40 and APOE were known to be linked to Alzheimer's disease [23]–[26]. Finally, longitudinal analysis also detected another significant SNP rs439401, which also belongs to gene APOE and is very close to SNP rs4420638; SNP rs4420638 was shown to be related to late on-set Alzheimer's disease [27], [28], dyslipidemia [29], schizophrenia [30], myocardial infarction [31] and psychological stress [32].

In addition, longitudinal analysis identified other four marginally significant SNPs that were missed by cross-sectional analysis at the significance level 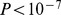: rs1800627, rs11762610, rs4902433 and rs439401 on chromosomes 4, 7, 14 and 19 respectively. On the other hand, at the same significance level, cross-sectional analysis, but not longitudinal analysis, uncovered two marginally significant SNPs rs2931352 and rs11875359. Since these SNPs were identified under a less stringent significance level, they are only suggestive and need to be further replicated and validated.

In addition to using the Bonferroni method to control the family-wise error rate, we also applied the false discovery rate (FDR) method [33] to adjust for multiple comparisons, as implemented in R function p. adjust (). The numbers of the significant SNP-phenotype associations at the various cut-offs of FDR are shown in Table 7. It is clear that at any given FDR level, longitudinal analysis identified a larger number of significant SNP-phenotype associations than that of cross sectional analysis. Note that, although an FDR (or q-value) is monotonic with the original unadjusted p-value, their relationship is not linear, which explains why at the FDR cut-offs of 0.035, 0.0011 and 0.0006, we reached the same numbers of the significant associations for longitudinal analysis, but not for cross sectional analysis, as those at the family-wise error rates of 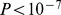, 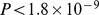 and 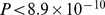, respectively.

**Table 7:** The numbers of the significant SNP-phenotype associations at various levels of false discovery rate (FDR).

FDR	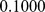	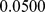	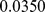	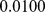	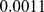	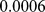
Longitudinal	112	90	83	64	46	43
Baseline	5	5	5	5	3	3

### Genome-wide association testing: more details with some phenotypes

**Figure 2:**
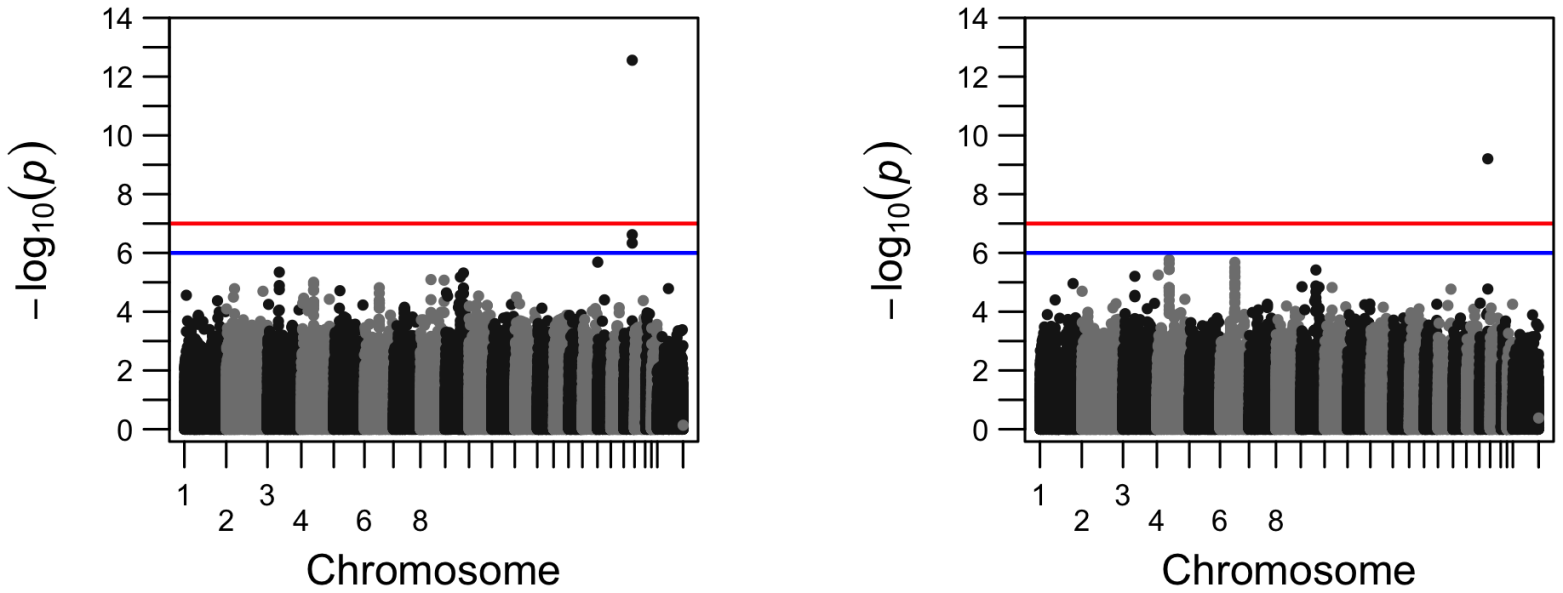
Comparison of the Manhattan plots for genome-wide p-values for phenotype left hippocampus volume from longitudinal analysis (left) and from cross-sectional analysis (right); SNP rs429358 is not included due to its small p-value.

**Figure 3:**
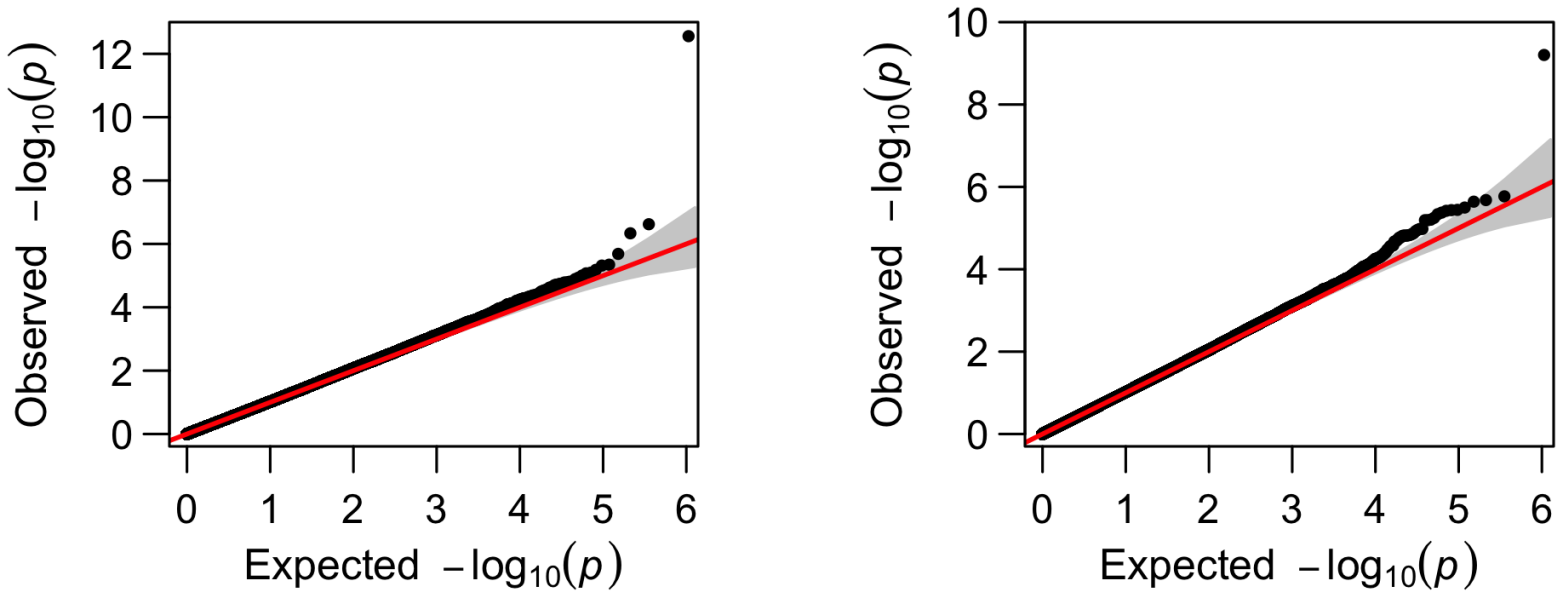
Comparison of the Q-Q plots for genome-wide p-values for phenotype left hippocampus volume from longitudinal analysis (left) and from cross-sectional analysis (right); SNP rs429358 is not included.

**Figure 4:**
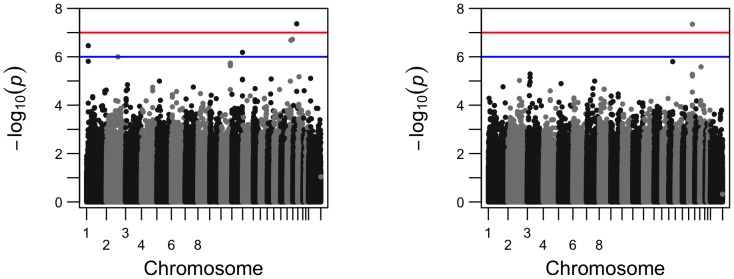
Comparison of the Manhattan plots for genome-wide p-values for phenotype volume of right inferior lateral ventricle from longitudinal analysis (left) and cross-sectional analysis (right); SNP rs429358 is not included.

**Figure 5:**
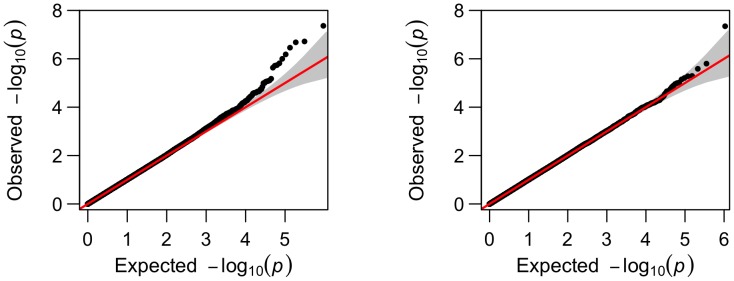
Comparison of the Q-Q plots for genome-wide p-values for phenotype volume of right inferior lateral ventricle from longitudinal analysis (left) and from cross-sectional analysis (right); SNP rs429358 is not included.

To visualize how the number of the significant SNPs increased from cross-sectional analysis to longitudinal analysis, we present their comparisons in Manhattan plots and Q-Q plots for two phenotypes, left hippocampus volume and right inferior lateral ventricle volume (Figures 2–5). For the volume of left hippocampus, 4 SNPs passed the significance level of 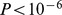 from longitudinal data analysis; in contrast, only 2 SNPs survived the threshold in cross sectional analysis. For volume of inferior lateral ventricle, there were respectively 6 and 2 SNPs passed the significance threshold of 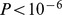 from longitudinal analysis and cross-sectional analysis.

### Population stratification

**Figure 6:**
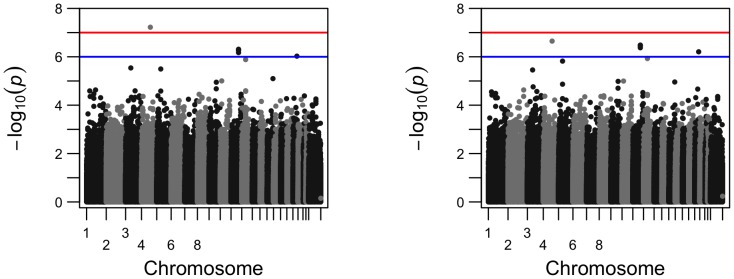
Comparison of the Manhattan plots without (left) or with (right) top 10 PCs.

**Figure 7:**
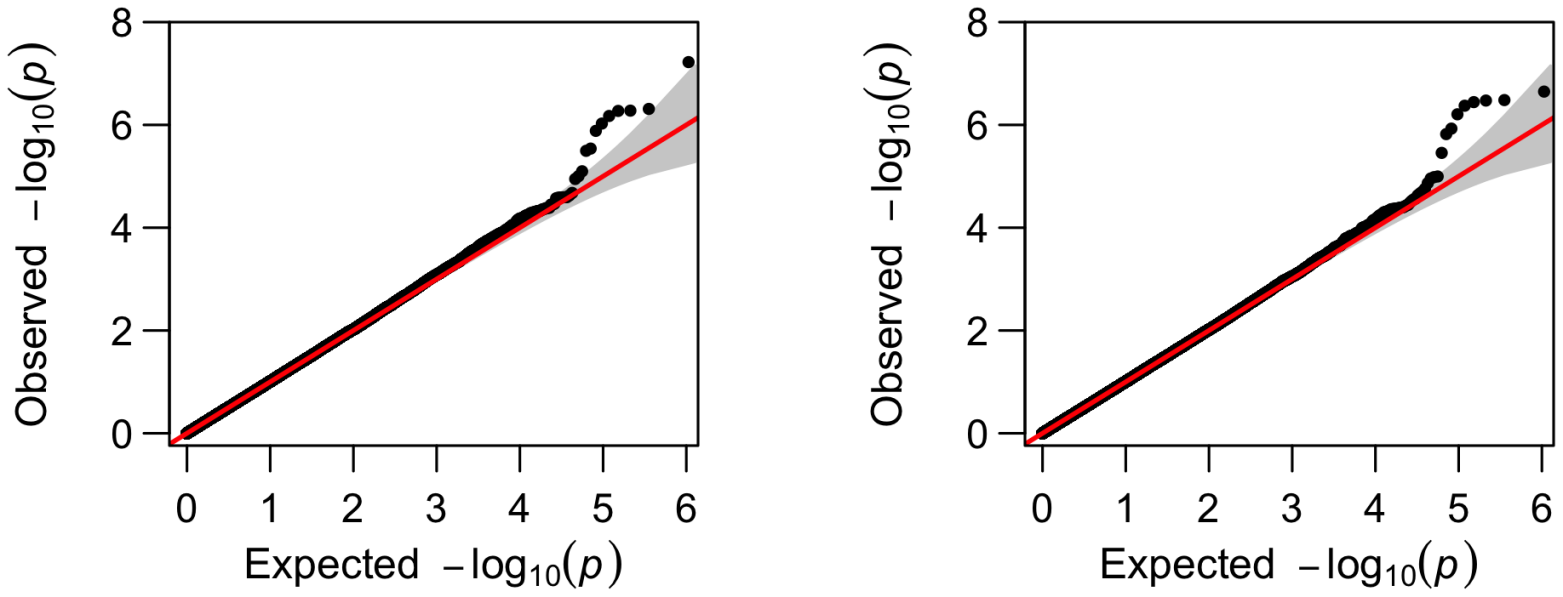
Comparison of the Q-Q plots without (left) or with (right) top 10 PCs.

To explore the possible existence of population stratification, we applied the principal component (PC) method [34]. Specifically, we first randomly selected 100,000 SNPs across the genome, then extracted the top ten PCs to be included as covariates in the linear mixed-effects model. For comparison, the Manhattan plots of the genome-wide p-values for phenotype volume of right amygdala are shown in Figure 6, while the Q-Q plots are in Figure 7. It is clear that whether or not to adjust for the top 10 PCs hardly made any difference to the overall results. Furthermore, we also calculated the genomic inflation factor 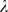 [35] in longitudinal data analysis. For phenotype volume of right amygdala, the inflation factor (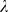) was estimated as 1.007 with the top 10 PCs, compared to 1.010 without PCs. A systematic examination of the inflation factors for all the 56 phenotypes without adjustment for PCs was also conducted: the estimated inflation factors ranged from 0.986 to 1.025 with mean 1.010 and standard error 0.014. Hence, in agreement with [4], there was no strong evidence for population stratification that would have questioned the validity of the genome-wide association results presented earlier.

### Alternative approaches to longitudinal analysis

**Figure 8:**
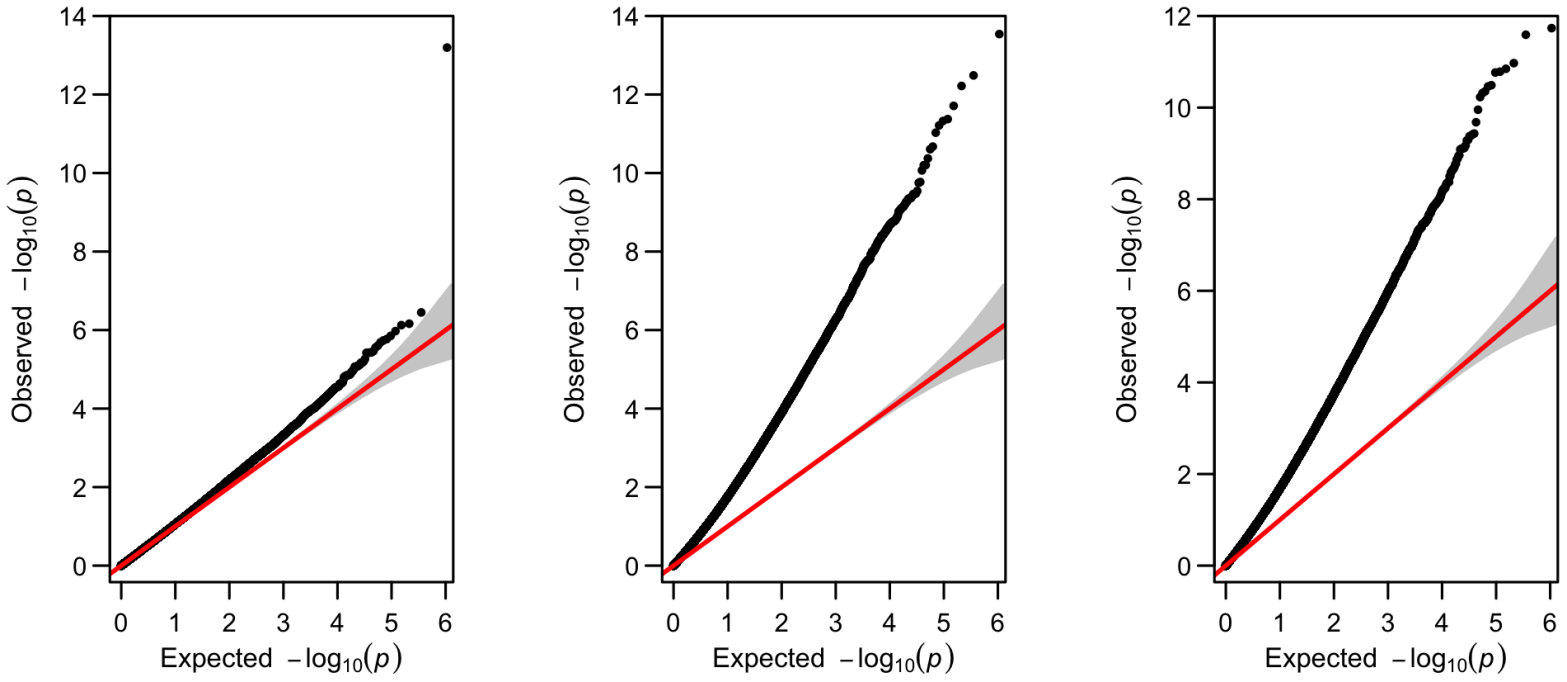
The Q-Q plots for genome-wide p-values for phenotype left hippocampus volume from longitudinal analysis based on (a) GEE with the sandwich covariance estimator (left, inflation factor  = 1.070), (b) GEE with the model-based covariance estimator (middle, inflation factor  = 2.077), and (c) linear mixed model with only a random intercept term (right, inflation factor  = 1.976).

We also applied a simpler linear mixed model (3) with only a random intercept term, and the corresponding GEE marginal model with a compound symmetry (CS) matrix as the working correlation structure. The results for phenotype left hippocampus volume are shown in Figure 8. Note that the results for the random-intercept model (3) and the GEE model with the model-based covariance matrix were very similar, as expected; both yielded severely inflated false positive rates with their estimated inflation factors much larger than 1. As analyzed in the Methods section, the problem was likely due to the mis-specification and thus under-estimation of the phenotype variances. In contrast, the GEE model with the sandwich estimator gave a much better controlled inflation factor, which however was still larger than that from the earlier linear mixed model (2); it could be due to the stronger “missing completely at random” assumption required by GEE, which might be violated here.

To establish the superiority of model (2) over model (3), we conducted the LRT on the null hypothesis 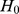: 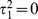 with phenotype left hippocampus volume and each of the 10196 SNPs on chromosome 19. The null hypothesis was rejected each time; the LRT statistics were large, ranging from 228.9 to 289.2 with a median of 279.9.

### Simulation results

When simulated phenotypic data were generated from model (2), as shown in Table 8, it was confirmed that only LME-RSI, GEE-Robust and Baseline could satisfactorily control the Type I error, while the other two could not due to their use of mis-specified models. Note that a difference between the real data and simulated data was that there were no missing phenotypes in the latter. For power, as expected, the two methods for longitudinal analysis, LME-RSI and GEE-Robust, were almost equally powerful, both more powerful than cross-sectional analysis.

**Table 8:** Simulation results at significance level 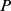 with different methods for phenotypic data generated from model (2).

Type I Error						
Model	rs2075650			rs439401		
	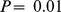	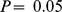	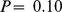	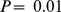	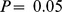	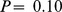
LME-RSI	0.007	0.044	0.087	0.007	0.039	0.097
LME-RI	0.071	0.177	0.258	0.090	0.190	0.276
GEE-Robust	0.008	0.045	0.089	0.008	0.045	0.106
GEE-Naive	0.082	0.189	0.257	0.102	0.191	0.286
Baseline	0.006	0.042	0.084	0.006	0.059	0.112

LME-RSI: a linear mixed-effects model with random slope and intercept; LME-RI: a linear mixed-effects model with only a random intercept term; GEE-Robust: GEE with the sandwich covariance estimator; GEE-Naive: GEE with the model-based covariance estimator; Baseline: a linear model at the baseline testing for the main effects of an SNP.

When simulated phenotypic data were generated from model (3), as shown in Table 9, the Type I error rates were generally controlled, though GEE-Naive might be too conservative. In terms of power, it is obvious that again longitudinal methods outperformed the cross sectional analysis of only the baseline data. Note that, since the CS working correlation structure used in GEE was correct, GEE-Naive also performed well. Most interestingly, though LME-RSI was fitted to a larger model (2) covering the true model (2) used in LME-RI, the power loss of LME-RSI was negligible when compared with LME-RI, showing the robustness of using model (2).

**Table 9:** Simulation results at significance level 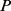 with different methods for phenotypic data generated from model (3).

Type I Error												
Model	rs2075650				rs439401				rs429358			
	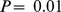	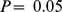	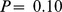	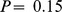	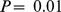	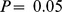	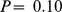	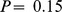	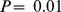	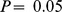	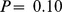	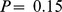
LME-RSI	0.006	0.038	0.079	0.136	0.007	0.043	0.096	0.143	0.007	0.043	0.097	0.140
LME-RI	0.006	0.039	0.079	0.135	0.008	0.043	0.096	0.147	0.007	0.044	0.096	0.141
GEE-Robust	0.004	0.040	0.088	0.135	0.007	0.052	0.104	0.148	0.009	0.045	0.100	0.145
GEE-Naive	0.000	0.012	0.031	0.043	0.001	0.010	0.033	0.059	0.001	0.010	0.023	0.044
Baseline	0.005	0.048	0.081	0.124	0.008	0.056	0.113	0.160	0.007	0.041	0.096	0.149

## Discussion

We have conducted a genome-wide association scan on each SNP with each of 56 regional imaging phenotypes utilizing the ADNI-1 data. By taking advantage of the existing longitudinal imaging phenotypes, we have illustrated the power gains from longitudinal analysis of longitudinal phenotypes measured at multiple time points over cross-sectional analysis of only the baseline phenotypic data. In particular, application of a linear mixed-effects model to longitudinal phenotypic data identified a much larger number of SNP-phenotype associations, at both a genome-wide and other less stringent significance levels. The advantage of longitudinal analysis is expected due to its use of more data, compared to only the baseline data in cross-sectional analysis. Note that here our goal is, as usual, to identify genetic variants associated with a phenotype in whatever way. We also note that our longitudinal model is related to, but different from, modeling gene-age interactions [36]: in the mean model (4), there is no SNP-baseline age interaction. If desired, as an alternative approach one can model SNP-age interaction directly. Since almost all existing GWAS analyses of the ADNI-1 data are based on the baseline data with only few exceptions [37]–[39], we hope that our current study will help reinforce the message on the preference of longitudinal analysis over cross-sectional analysis. This issue will become even more compelling as more longitudinal phenotypic data, e.g. various neuroimaging phenotypes from ADNI-GO and ADNI-2, are being or will be collected.

We have only applied single SNP-based analyses, while there is increasing evidence of possible power gains with SNP-set analyses in cross-sectional studies [9], [40]. However, it remains to be done to extend some powerful SNP-set methods to mixed-effects models with longitudinal data, such as the variance-component or kernel methods, similarity-based tests and others [41]–[46]. Or, instead of controlling family-wise Type I error rate as approached here, one may apply some new methods to control false discovery rate (FDR) [47]. Furthermore, with the availability of DNA sequencing data, it will be useful to develop and apply new statistical tests to detect rare variant associations with longitudinal phenotypes, again based on some extensions of the methods for cross-sectional data [46], [48]–[53]. More generally, as pointed out by Lindquist [54], “Imaging genetics promises to be an important topic of future research, and to fully realize its promise, novel statistical techniques will be needed.” These are all interesting topics to be investigated.
